# Chronically stressed male and female mice show a similar peripheral and central pro-inflammatory profile after an immune challenge

**DOI:** 10.1371/journal.pone.0297776

**Published:** 2024-02-21

**Authors:** Mariella Bodemeier Loayza Careaga, T. John Wu

**Affiliations:** 1 Department of Gynecologic Surgery and Obstetrics, Uniformed Services University of the Health Sciences, Bethesda, Maryland, United States of America; 2 Henry M. Jackson Foundation for the Advancement of Military Medicine, Inc., Bethesda, Maryland, United States of America; 3 Program in Neuroscience, Uniformed Services University of the Health Sciences, Bethesda, Maryland, United States of America; Universidade de Sao Paulo, BRAZIL

## Abstract

Although acute stressors are known for stimulating the production of glucocorticoids and pro-inflammatory cytokines in rodents, the effects of chronic stressors on cytokine levels and the activation of the hypothalamic-pituitary-adrenal (HPA) axis, especially in response to a subsequent challenge, are less clear. In this study, male and female mice were exposed to 6 weeks of chronic variable stress (CVS) and the peripheral and central levels of IL-1β, IL-6, and TNF-α, as well as the HPA axis reactivity, were measured after an acute injection of LPS. The findings indicate that the pro-inflammatory profile in the plasma, regardless of stress exposure, was similar between male and female animals, whereas there was a region-, sex-, and stress-dependent pattern in the brain. Exposure to chronic stressors blunted the HPA reactivity to the LPS challenge, indicating a modulatory effect on the stress axis responsiveness.

## Introduction

The physiological stress response is a set of mechanisms that allows the organism to adapt to environmental changes [1]; yet, persistent exposure to stressors is linked to dysregulation of the hypothalamic-pituitary-adrenal (HPA) axis [2, 3] and greater risk of developing several health conditions, including cardiovascular disease [4], metabolic disruption [5] and psychiatric disorders [6, 7].

Glucocorticoids (corticosterone, CORT, in rodents and cortisol in humans) are released by the adrenal glands in response to exposure to stressors and are known for modulating the immune system [8]. Previous studies in rodents showed that acute stressors increase the expression of peripheral and central inflammatory cytokines such as interleukins 1β (IL-1β) and 6 (IL-6) [9, 10] and potentiates the pro-inflammatory response to a subsequent immune stressor [11–14]. These effects of stress on the inflammatory response are dependent on the timing of CORT or stress exposure. CORT or exposure to an acute stressor increases pro-inflammatory cytokines when prior to an immune challenge [11, 15], whereas CORT reduces the expression of pro-inflammatory cytokines in the brain when given post-LPS injection [15]. Additionally, previous studies showed that the stress-induced potentiation of pro-inflammatory responses to an immune challenge in the hippocampus are mediated by glucocorticoids in both male and female rats [16]. These steroid hormones, in turn, act on the microglia to induce their neuroinflammatory priming effects on male, but not female rats [13, 16], suggesting that although both sexes show stress-induced potentiation to an immune challenge, this process occur through sex-specific mechanisms.

Contrary to acute stressors, the impact of chronic exposure to stressors on the pro-inflammatory profile and HPA axis responsiveness is varied. For instance, while exposure to chronic mild stressors reduces IL-6 levels in the liver and increases the hypothalamic expression of IL-6, but not IL-1β, in female mice [17], chronic mild stressors stimulate the plasmatic and central production of IL-6, IL-1β, and tumor necrosis factor alpha (TNF-α) in male mice [18]. Male rats previously exposed to chronic cold show enhanced secretion of CORT, adrenocorticotropic hormone (ACTH), and peripheral and central pro-inflammatory cytokines in response to a subsequent stressor [19, 20], whereas chronic mild stressors attenuate CORT secretion, as well as IL-1β and TNF-α responses in the prefrontal cortex of male rats [21].

To date, most preclinical studies investigating the relationship between chronic exposure to stressors and immune response were either conducted on a single sex or, when including both sexes, analyzed chronic stress effects on baseline parameters without challenging the animals with a subsequent stressor [17, 18, 20–26]. Considering sex as a variable when studying the chronic stress-immunity dyad is important as previous studies identified sex differences in the HPA axis activity at baseline and in response to stress. For instance, female rodents have higher resting and stress-induced CORT [27–29] and ACTH [30] levels than males. Sex differences are also observed in the expression of neuropeptides in the paraventricular nucleus of the hypothalamus [31], as well as in the mRNA levels of key regulators of the HPA axis activity [32]. Moreover, sex differences in the priming effects of acute stressors on the neuroinflammatory responses have been previously reported, with prior exposure to stress sensitizing the cytokine levels in the microglia of males, while stimulating serum IL-1β production in females after an immune challenge [16]. As stress-related disorders are more prevalent in women than in men [33], assessing the immune consequences of chronic exposure to stressors in females may be informative as we develop strategies to mitigate the deleterious consequences of chronic stressors on human health.

Thus, the aim of this study was to investigate the effects of a chronic variable stress (CVS) protocol on the peripheral and central pro-inflammatory profile as well as on the reactivity of the HPA axis in response to a subsequent immune stressor in both male and female mice. As a stress paradigm designed to study the effects of prolonged exposure to stressors, the CVS procedure employs a battery of stressful stimuli that are randomly introduced for different durations over weeks or months. The unpredictable and variable nature of the paradigm minimizes the adaptation of the physiological stress response often seen following repeated presentation of homotypic stressors [34], while inducing a range of behavioral, neuroendocrine, and neurobiological changes in rodents [35–42]. We measured the levels of IL-1β, IL-6, and TNF-α, three key pro-inflammatory cytokines [43], in both plasma and brain tissue as peripheral and central cytokines levels may vary upon exposure to chronic stressors [17, 19–21]. Also, by performing the experiments simultaneously in both sexes, we designed an experimental condition that allowed us to uncover potential sex differences while controlling other experimental variables that often limit the direct comparison between preclinical chronic stress studies.

## Experimental procedures

### Animals

Male and female C57BL/6J mice (Jackson Laboratory, Bar Harbor, ME) were used in the experiments. Animals were 7 weeks old at the beginning of the experiments, and were maintained on a 12h: 12h, light: dark cycle (lights on at 01:00 am) under controlled temperature (22–25°C), humidity (30–70%) and *ad libitum* access to food and water. Upon arrival, mice were group housed with same-sex cage mates (2–4 animals/cage) and were given 10 days of acclimation to the facilities prior to experimentation. The animals were handled for 2 min on three different days during acclimation, and all animal cages contained nesting material and rodent enrichment devices (plastic tunnels or igloos). During the CVS period, mice were handled weekly for cage change and body weight measurement. Animals were randomly assigned to one of 2 treatment conditions based on a 2X2X2 factorial design consisting of chronic variable stress (CVS or No CVS), sex (male or female) and LPS administration (LPS or vehicle). All experiments were approved by the Uniformed Services University of the Health Science animal use and care committee.

### Chronic Variable Stress paradigm (CVS)

Animals underwent 6 weeks of chronic variable mild stress. Each day, mice were exposed to 1 or 2 different stressors (AM stressor between 7:00–12:30; PM stressor between 13:00–17:00) in a semi-random manner. Body weight was measured two times per week before the morning stressor. Animals were exposed to the stressors in a separated room, and control mice remained undisturbed in the colony room. The stressors in the CVS paradigm were the same used on a previous work from our group [32], and included a selection of stressors that varied in nature and duration. During stress exposure, the enrichment devices in the animal’s home cages were removed.

### Tissue and blood collection

**Fig 1:**
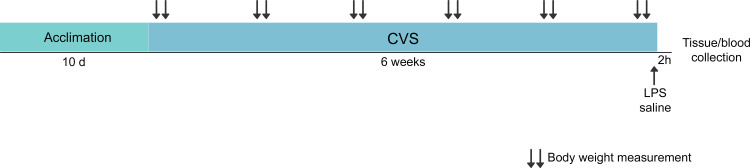
Experimental timeline.

Two hours after the end of the last overnight stressor, mice received an acute injection of LPS (Lipopolysaccharides from Escherichia coli O111:B4; Sigma-Aldrich, cat. #L2630; 0.2 mg/Kg dissolved in 0.9% saline) or vehicle (0.9% saline) intraperitoneally in a volume of 0.1 mL. The animals were then returned to their home cages and anesthetized by carbon dioxide inhalation and rapidly decapitated two hours after treatment (Fig 1). Both LPS dosage and euthanasia time were selected based on previous rodent studies [13, 20, 44, 45]. Brain and trunk blood were immediately harvested. Brains were flash frozen in 2-methyl-butane on dry ice and stored at -80°C until use. Trunk blood was collected in 1.5 mL EDTA-coated tubes and kept under constant rotation at room temperature until centrifugation. The adrenal glands, thymi and spleens were also collected from a subset of animals (64 mice). The tissue samples were quickly dissected, collected into 1.5 mL tubes and frozen in dry ice.

### Tissue and blood processing for cytokine quantification

Brain samples were sectioned into 1mm coronal sections, and the prefrontal cortex (PFC), hippocampus and hypothalamus were microdissected using a 1 mm punch. All microdissections were performed following the coordinates of the mouse brain atlas [46]. The PFC microdissections included the infralimbic (IL), pre-limbic (PL) and anterior cingulate (AC) regions (from bregma 1.98 mm to 1.34 mm). For the hippocampus, tissue was collected from both the dorsal and ventral hippocampi (from bregma -1.22 mm to -3.40 mm). The hypothalamus microdissections included different nuclei within bregma -0.58 mm to -1.34 mm coordinates.

Brain samples were homogenized in sterile PBS with protease inhibitor cocktail (10 to 1 v/w, 1 mL for 100 mg of tissue; cat. # P2714-1BTL; Sigma-Aldrich, St. Louis, MO) using a manual single channel pipette. Tissue lysates were then centrifuged at 16,000 x g for 10 min at 4°C, and the supernatant was collected and stored at -80°C until assayed. Total protein concentration was determined using Pierce’s BCA assay (Pierce BCA Protein Assay Kit, cat. #23225; Thermo Fisher, Waltham, MA), and predetermined total protein quantities were used for measuring brain cytokine levels using the mouse high sensitivity T Cell Magnetic Bead Panel (Milliplex; Cat. # MHSTCMAG-70K; MilliporeSigma, Burlington, MA). Briefly, 25 μL of the samples were loaded into the plate and incubated for around 17 h with 25 μL of mixed magnetic beads. The plate was washed 5 times with the kit’s wash buffer, and then incubated for 1 h with the provided detection antibody, followed by a 30-minute incubation with streptavidin-phycoerythrin. After an additional washing step, the beads were resuspended with 150 μL of wash buffer and read in a Bio-Plex 200 system (Bio-Rad Laboratories, Hercules, CA).

To assess the cytokine levels in the periphery, plasma was separated from blood by centrifugation at 2,000 x g for 15 min at 4°C no longer than 30 minutes after blood collection. Peripheral cytokines levels were measured using the mouse high sensitivity T Cell Magnetic Bead Panel following the steps described above. For plasma cytokines quantification, samples were diluted 1:2 before assaying. All samples were analyzed in duplicates.

### Corticosterone assay

Trunk blood was collected in EDTA-coated tubes and plasma was obtained for corticosterone (CORT) assessment. An enzyme-linked immunosorbent assay (ELISA) was used to measure CORT per manufacturer’s instructions (DetectX Corticosterone; cat. # K014-H5; Arbor Assays, Ann Arbor, MI). Samples were diluted to a final concentration of 1:100 and analyzed in duplicates. Changes in binding were determined in a plate reader, and absorbance was read at 450 nm. The average intra-assay %CV ranged from 2.6 to 2.9, and the mean inter-assay %CV was 2.7. All values were obtained by comparison to a standard curve ranging from 39.06 to 10,000 pg/mL.

### Statistical analysis

Primary outcome variables were analyzed using repeated measures, one- and three-way analysis of variance (ANOVA) with CVS, treatment, and sex as between-subjects factors. For the repeated measures analyses, Mauchly’s Sphericity test was used, and the Greenhouse-Geisser correction was applied when sphericity was not met. Bonferroni post hoc was applied in all pairwise comparisons. The Grubbs’ test was used to identify possible outliers, and these values were excluded from the final data analyses. The study involved a total of one hundred and thirty-four mice. In the analysis of the peripheral tissues, we excluded 3 mice for the adrenal weight assessment and 1 mouse from spleen measurement due to missing data and outlier analysis. In the CORT analyses, three mice were excluded (1 at baseline; 2 at post-CVS) for being classified as outliers, and seven mice due to missing plasma samples or technical issues during the assay. Initially, 120 animals were included in the plasma cytokines analyses. However, after accounting for missing data and outliers, the final analysis included 112 mice for IL-1β, 68 for TNF-α, and 83 for IL-6. For the assessment of cytokines in different brain regions, we initially included 112 mice. However, due to issues with tissue collection, cytokine measurement, and the exclusion of outliers, the final analyses included 94–95 mice for the hypothalamus, 79–106 mice for the hippocampus, and 72–77 mice for the PFC. All data analysis was performed using IBM SPSS 28 (IBM, Armonk, NY) and represented by the mean and the standard error of the mean. The p-value for all statistical analyses, including Grubb’s test, was set to 0.05.

## Results

### The CVS impacts body weight gain in a sex-dependent manner while LPS treatment induces tissue-specific weight changes

**Fig 2:**
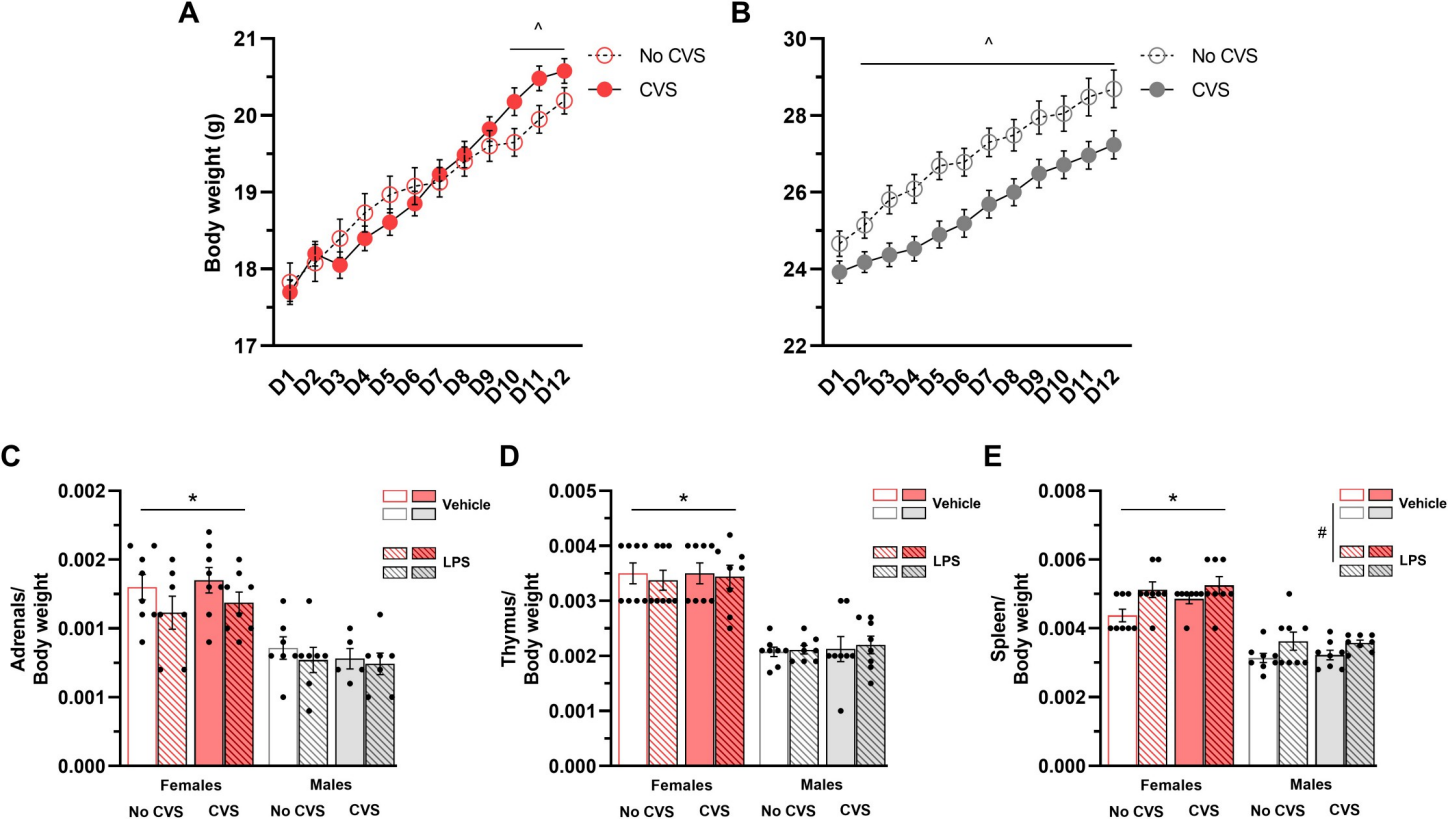
Sex differences on the effects of the CVS protocol and LPS challenge on body weight and tissue masses. Six weeks of CVS induced body weight gain in female (A) but not in male (B) mice. Female animals also had heavier adrenals (C), thymi (D), and spleens (E) compared to their male counterparts, and LPS-treated animals exhibited heavier spleens than saline-treated mice. Data are presented as mean ± SEM. Body weight data was analyzed using one-way repeated measures ANOVA and tissue weight was analyzed using three-way ANOVA (n = 5–8 mice/group), p < 0.05. # Different from saline-treated mice; * Different from males; ^ Different from non-CVS groups.

Body weight was measured twice a week throughout the CVS period to assess the effects of exposure to chronic stressors on body weight gain. Over the 6 weeks of assessment, both non-stressed and stressed females gained weight (F_(4.6,267.6)_ = 249.80, P < 0.05). Yet, starting at D10 (5^th^ week of CVS), stressed females gained more weight than their non-stressed counterparts (F_(4.6,267.6)_ = 9.94, P < 0.05; Fig 2A). In males, both CVS-exposed and control mice gained weight over the 6 weeks of assessment (F_(2.03,117.76)_ = 328.40, P < 0.05). However, CVS-exposed mice gained less weight than non-stressed animals (F_(1,58)_ = 7.65, P < 0.05) and effect observed from D2 to D12 of measurement (F_(2.03,117.76)_ = 4.60, P < 0.05; Fig 2B).

The normalized weight of the adrenal glands, thymus and spleen were also assessed. For the adrenals, a main effect of sex was found, and females had heavier adrenal glands than males (F_(1,49)_ = 47.87, P < 0.05; Fig 2C). A similar effect was observed on the thymus, and females showed heavier thymi compared to their male counterparts (F_(1,56)_ = 117.63, P < 0.05; Fig 2D). For the normalized spleen weight, a three-way ANOVA revealed main effects of Sex (F_(1,55)_ = 127.95, P < 0.05) and Treatment (F_(1,55)_ = 13.73, P < 0.05). Females showed heavier spleens compared to males, and LPS-treated mice had heavier spleens than saline-injected animals (Fig 2E).

### LPS-induced corticosterone increase is not altered by CVS exposure

**Fig 3:**
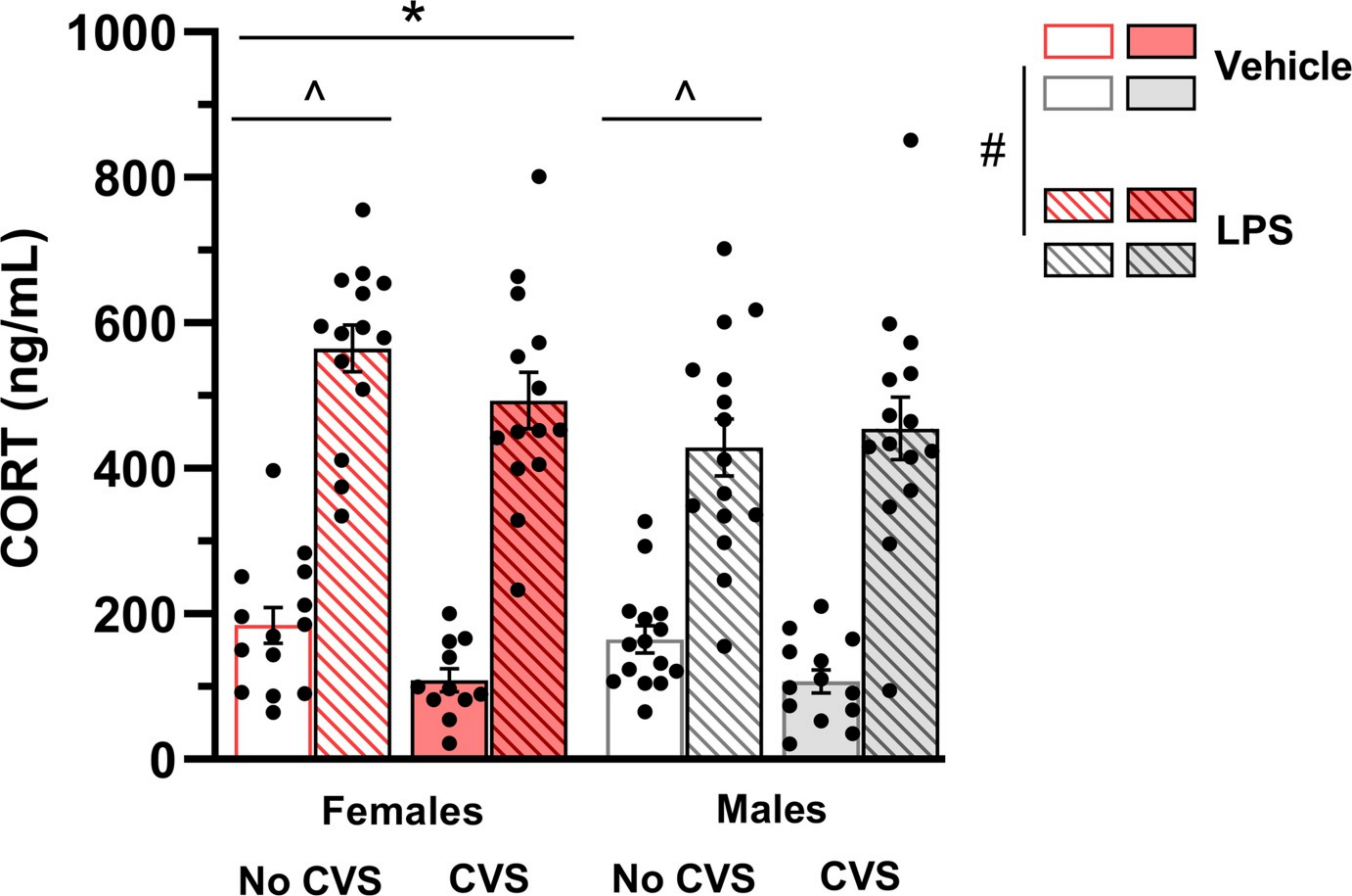
CVS paradigm does not affect LPS-induced increase on corticosterone (CORT) levels. Plasma levels of CORT were measured after CVS and LPS injection. Three-way ANOVA. Data are presented as mean ± SEM, p <0.05. n = 11–15 mice/group. # Different from saline-treated mice; * Different from males; ^ Different from non-CVS groups.

CORT plasma levels were measured after CVS and LPS injection to assess the impact of exposure to chronic stressors and an immune challenge on circulating glucocorticoids concentrations. To establish a baseline level for CORT, a subset of non-stressed, untreated mice was anesthetized with carbon dioxide, euthanized, and their trunk blood was collected during CORT nadir. No sex differences were detected on the baseline levels of CORT (S1 Fig; F_(1,11)_ = 3.17, P = 0.1). In stress-exposed and control animals injected with LPS or saline, CVS did not alter the LPS-induced CORT response, and CORT levels were significantly higher in mice injected with LPS compared to animals treated with saline (F_(1,103)_ = 237.66, P <0.05). When the overall CORT concentration in female mice was compared to male animals, a sex difference was observed and female mice had higher CORT levels than male counterparts (F_(1,103)_ = 4.78, P = 0.03). Lastly, stress-exposed groups, regardless of treatment condition, had lower CORT levels compared to non-stressed groups (F_(1,103)_ = 4.03, P = 0.05; Fig 3).

### The CVS paradigm does not impact the peripheral pro-inflammatory profile after an immune challenge

**Fig 4:**
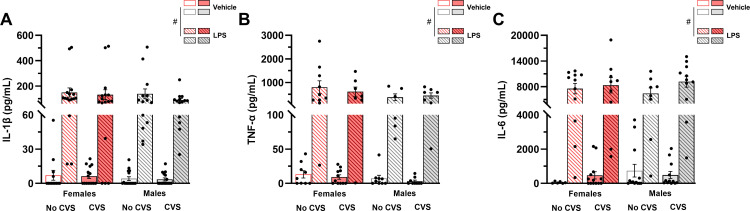
The CVS paradigm does not alter the pro-inflammatory cytokines in the plasma after an immune challenge. The levels of IL-1β, TNF-α and IL-6 were assessed in plasma 2 hours after LPS injection. Three-way ANOVA. Data are presented as mean ± SEM, p < 0.05. n = 6–15 mice/group. # Different from saline-treated mice.

The levels of IL-1β, TNF-α and IL-6 were measured in the plasma after LPS injection to evaluate the effects of the CVS paradigm on the peripheral inflammatory response. LPS treatment induced a robust increase in the levels of IL-1β (F_(1,104)_ = 47.91, P < 0.05; Fig 4A), TNF-α (F_(1,60)_ = 31.78, P < 0.05; Fig 4B) and IL-6 (F_(1,75)_ = 113.06, P < 0.05; Fig 4C) in both male and female mice. CVS exposure did not alter the LPS-induced cytokine increase as both non-stressed and stressed groups showed similar levels of pro-inflammatory cytokines in the plasma.

### The effects of CVS exposure and bacterial endotoxin challenge are dependent on the brain structure

To further investigate the effects of CVS and the immune challenge on the pro-inflammatory profile in the brain, the levels of IL-1β, TNF-α and IL-6 were assessed in the hippocampus, prefrontal cortex, and hypothalamus.

**Fig 5:**
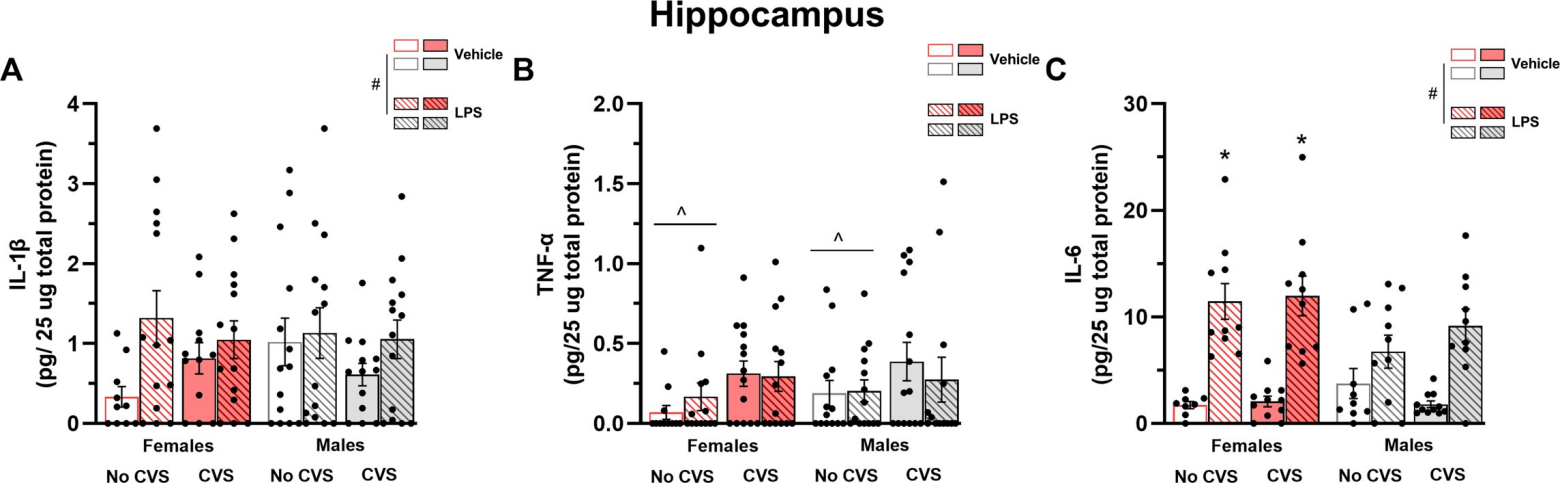
Sex and CVS dependent effects on the hippocampus are cytokine specific. Brains were collected for cytokine assessment 2 hours after LPS injection. Three-way ANOVA. Data are mean ± SEM, p < 0.05. n = 10–14 mice/group. # Different from saline-treated mice; * Different from males; ^ Different from non-CVS groups.

In the hippocampus, mice treated with LPS showed increased levels of IL-1β (F_(1,97)_ = 5.78, P = 0.02) and IL-6 (F_(1,71)_ = 65.70, P < 0.05) but not TNF-α (P > 0.05) (Fig 5). A sex and treatment interaction was also found for IL-6 (F_(1,71)_ = 6.28, P = 0.02), in which LPS-treated female animals had higher levels of IL-6 compared to LPS-treated male mice (Fig 5C). Exposure CVS only affected the levels of TNF-α (F_(1,98)_ = 5.62, P = 0.02), and stress-exposed groups showed increased TNF-α levels than their non-stressed counterparts (Fig 5B).

**Fig 6:**
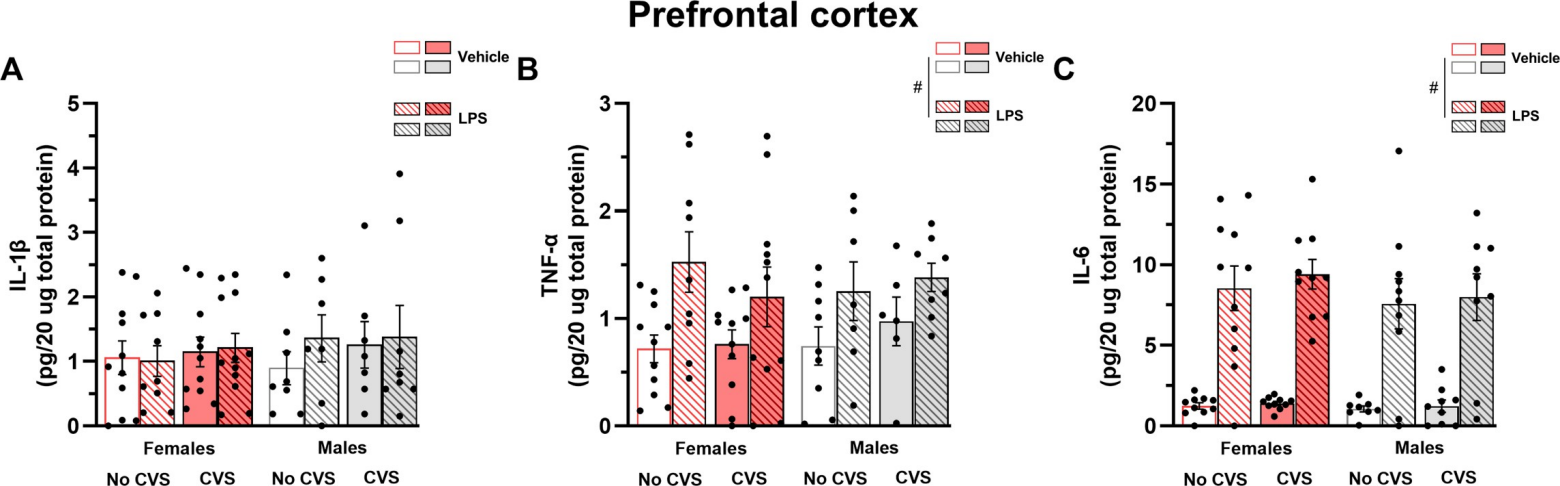
The CVS paradigm, as well as sex, does not impact the levels of IL-1β, TNF-α and IL-6 in the prefrontal cortex. Brains were collected for cytokine assessment 2 hours after LPS injection. Data are mean ± SEM. Three-way ANOVA, p < 0.05. n = 6–12 mice/group. # Different from saline-treated mice.

In the PFC of both male and female mice, the LPS injection increased the levels of TNF-α (F_(1,64)_ = 12.49, P < 0.05) and IL-6 (F_(1,69)_ = 99.93, P < 0.05) but not IL-1β (P > 0.05) (Fig 6). Neither sex nor CVS exposure affected the levels of these pro-inflammatory cytokines.

**Fig 7:**
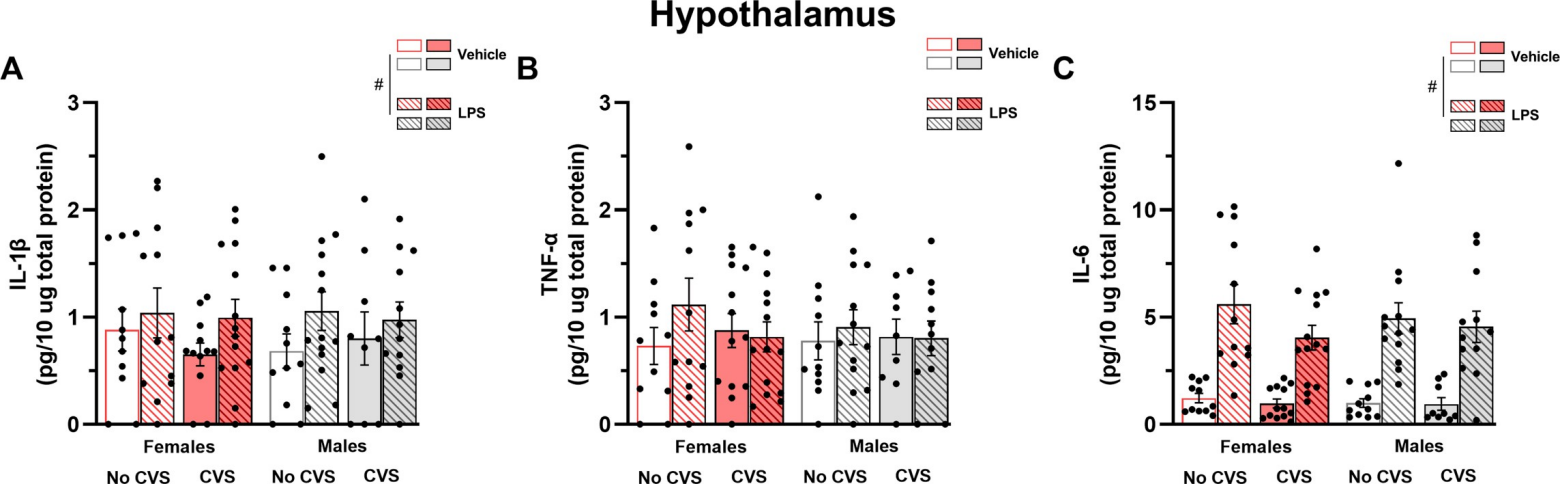
No effects of CVS or sex are detected on the levels of IL-1β, TNF-α and IL-6 in the hypothalamus. Brain tissue was collected 2 hours after LPS injection. Data are presented as mean ± SEM. Three-way ANOVA, p < 0.05. n = 9–14 mice/group. # Different from saline-treated mice.

The effects of LPS on the proinflammatory profile in the hypothalamus were similar to the one found in the hippocampus. In both female and male mice, LPS treatment increased the levels of IL-1β (F_(1,87)_ = 3.97, P = 0.05) and IL-6 (F_(1,87)_ = 83.13, P < 0.05) but not TNF-α (P > 0.05) (Fig 7). Neither sex nor CVS exposure impacted the cytokines levels in this brain region.

## Discussion

In this study, we evaluated the effects of a CVS protocol on the peripheral and central levels of pro-inflammatory cytokines, as well as the reactivity of the HPA axis, in male and female mice. Our findings suggest that sex differences on pro-inflammatory consequences of chronic stressors are limited, showing a region-, sex-, and stress-dependent pattern in the brain. Though CVS exposure did not significantly alter the pro-inflammatory response of mice, it blunted the CORT response to the LPS challenge, indicating a modulatory effect on the stress axis responsiveness.

The physiological impact of CVS exposure in both sexes was assessed by measuring the animals’ body weight, as well as the adrenal, thymus, and spleen normalized weights. Starting at the first week of CVS, stress-exposed male mice gained less weight than their respective controls, whereas stress-exposed female mice increased their body weight over the last week of the protocol. The reduction in body weight gain observed in male animals is in line with previous findings showing attenuation of body weight gain in chronically stressed rodents [2, 17, 24, 40, 41, 47, 48]. Thought previous studies, including one from our laboratory, suggested that chronic stress attenuates body weight gain also in female rodents [17, 24, 32, 49], there is evidence that chronically stressed male and female mice might gain weight after chronic social defeat [50, 51], suggesting that the metabolic effects of stress exposure might be linked with the nature of the stressors regimen. Also, female mice showed increased intake of highly palatable food after 2 weeks of a CVS paradigm, revealing an increased sensitivity of food-seeking behaviors in females compared to males [52]. Thus, it is possible that in our study, CVS-exposed female mice may have increased their food consumption towards the end of the stress paradigm, which could have affected their body weight during the last two weeks of the protocol. This hypothesis, however, warrants further investigation as we did not record the pellet consumption of the different groups in the present study.

The present CVS paradigm did not induce hypertrophy of the adrenal glands and involution of the thymus, in line with previous findings from our laboratory using the same protocol [32]. Hypertrophy of the adrenals and involution of the thymus are considered hallmarks of long-term stress exposure in rodents. Yet, some studies fail to report those changes in mice [48] or only show an effect of chronic stressors in one of those tissues [3, 40, 42, 53]. Taken together, these findings suggest that differences in the severity of the chronic stress protocol, animal species, and rodent sex might influence the impact of the stressors on those glands. Although the CVS paradigm did not affect the adrenals and thymus, a clear sex difference in the normalized weight of these tissues was observed and female animals had heavier adrenals and thymi than their male counterparts. This result indicates an innate difference in the weight of these glands when comparing both sexes [40].

We also assessed weight changes in the spleen after the acute immune challenge as this organ is an important secondary lymphoid organ that plays a role in the induction of an effective immune response. We observed a sex difference in the normalized weight of the spleen, and female mice had heavier spleens compared to male animals. A previous study revealed a sex- and age-dependent difference in the normalized weight of the spleen, with young and old female mice having heavier spleens than their respective male counterparts. These weight differences were accompanied by changes in the composition of immune cells, suggesting a correlation between alterations in spleen size and the frequency of these cells within the organ [54]. Building upon these previous findings, our current results suggest the possibility of a shift in the types and number of immune cells within the spleens of female mice, a change that could potentially impact the overall immune function of that organ. Moreover, LPS-treated mice had heavier spleens compared to their respective control groups. Acute bacterial endotoxin treatment stimulates the production of catecholamine and pro-inflammatory cytokines, as well as infiltration of monocytes and neutrophils into the spleen of rats [55]. These changes in the organ function, along with the recruitment of immune circulating cells into the spleen, might explain the increased normalized weight seen in the present study.

To assess chronic-stress immune consequences, we looked at three key pro-inflammatory cytokines (IL-1β, TNF-α, and IL-6), measuring their concentrations in the plasma and in three brain regions key for the regulation of the stress response. In plasma, LPS stimulated the production of all pro-inflammatory cytokines in both non-stressed and stressed mice. As the major component of the outer membrane of gram-negative bacteria, LPS is readily identified by circulating immune cells and triggers a robust peripheral pro-inflammatory response in rodents [18, 20]. Exposure to chronic stressors may affect the peripheral pro-inflammatory response, but this effect seems to be cytokine, tissue, and stress modality dependent. For instance, after a LPS challenge, chronic cold exposure sensitizes the plasmatic IL-6, but not IL-1β response [20], while repeated mild stressor exposure increases the expression of IL-1β in the liver [25] and repeated immobilization stimulates mRNA expression of splenic TNF-α and IL-6, but not IL-1β [55].

Our plasma cytokine measurements revealed no sex difference in the pro-inflammatory response after the immune challenge. Though previous rodent studies also investigated the effects of the exposure to chronic stressors on pro-inflammatory markers, many relied on a single-sex analysis or, when including both sexes, did not challenge the animals with a subsequent stressor [20, 24, 26]. Thus, by directly comparing male and female mice, our study provides relevant data on the lack of sex differences of CVS on the peripheral pro-inflammatory response.

The immune consequences of CVS exposure in the central nervous system were evaluated by measuring IL-1β, TNF-α, and IL-6 in the hippocampus, PFC, and hypothalamus. The hippocampus and PFC are critical for the regulation of the stress response [1, 56], while the hypothalamus is the location of the paraventricular nucleus (PVN), a key coordinator of the HPA axis response [57]. Though our peripheral pro-inflammatory profile revealed a consistent effect of the bacterial endotoxin challenge in all cytokines studied, the central effects were less consistent and revealed region, CVS, and sex dependent patterns. We observed increased levels of IL-6 in all brain regions; yet a sex dependent effect was only found in the hippocampus, where LPS-injected female mice showed higher IL-6 levels than their male counterparts. Upon bacterial endotoxin treatment, the expression of the IL-6 receptor, IL-6R, is up-regulated in many brain regions, including the hippocampus, cerebral cortex, and PVN [58]. As basal levels of IL-6 in the plasma, PFC, and hippocampus are similar between male and female mice [26], the present findings suggest that a sex-dependent effect on the IL-6 response arises in the hippocampus only upon an immune challenge. A sexual dimorphism in IL-6 levels could influence several physiological functions of the hippocampus. For instance, both deficiency or excess of IL-6 in the brain has been associated with impaired performance in learning and memory tasks in rodents [59], some of which rely on the hippocampal function [60]. Moreover, the impact of the exposure to chronic stressors on LPS-induced IL-6 levels seems to be regimen dependent, as exposure to chronic cold stressor sensitizes the central and peripheral IL-6 responses [20], whereas repeated social defeat attenuates PFC IL-6 response to LPS [22]. Thus, these findings, along with ours, indicate that characteristics of the stress regimen determine whether IL-6 response will be potentiated, attenuated, or unaltered after a subsequent immune stressor.

The current CVS protocol only affected the hippocampal TNF-α levels, increasing its expression in both stress-exposed male and female animals regardless of LPS treatment. The effects of the exposure to chronic stressors on hippocampal TNF-α levels might depend on the duration of the stressors’ regimen. For instance, ten days of exposure to mild stressors does not increase TNF-α mRNA expression in the mouse hippocampus [25]; however, nine weeks of exposure to unpredictable chronic mild stressors increases TNF-α protein levels in the same brain region [18]. These results, along with ours, suggest that the duration of the exposure to chronic stressors is key for stimulating TNF-α production in the hippocampus. The stress-induced increase in hippocampal TNF-α levels could influence how stress affects anxiety-like behaviors. Male mice exposed to an acute stressor showed elevated levels of TNF-α in the ventral hippocampus, concurrent with anxiety-like behavior. Abolishing TNF-α production in these animals mitigated the stress-induced anxiety changes [61]. Previous behavioral findings from our laboratory indicated that exposure to the present CVS protocol also induced anxiety-like changes in both male and female mice [32]. The current results, therefore, suggest a potential neuroimmune alteration underlying these phenotypical changes induced by chronic stress.

The effects of exposure to stressors on brain IL-1β were one of the first to be investigated in rodents. Previous studies suggested that acute stressors stimulate IL-1β production in brain regions such as the hypothalamus and hippocampus [9–11]. In the present study, we observed a region-dependent increase on IL-1β protein levels after the acute LPS challenge. The bacterial endotoxin treatment stimulated the production of IL-1β in the hippocampus and hypothalamus, but not PFC, of male and female mice. The present hypothalamic and hippocampal IL-1β increase is in line with previous findings showing higher IL-1β levels in response to LPS treatment [18, 20]. Though previous studies also suggested an increase of IL-1β mRNA and protein levels upon LPS injection in the PFC [18, 20, 22, 25], this stimulatory effect is not always reported [21]. These inconsistencies might be explained by differences in the LPS dose, as well as the time interval between gene or protein expression assessment and LPS injection. While a previous study using a 2-week long CVS protocol showed an attenuation of IL-1β mRNA levels in the PFC of CVS-exposed mice [21], the present findings are in line with other studies using chronic cold and social defeat which show no impact of chronic stressors on hypothalamic and PFC IL-1β levels [20, 22].

Glucocorticoids, the main mediators of the physiological stress response, are potent regulators of the inflammatory response [8], and rodents chronically stressed often show changes in baseline and stress-induced CORT levels [3, 32, 39]. Thus, we evaluated the impact of the current CVS protocol on the HPA axis responsiveness to a novel, acute immunological challenge. Both male and female mice, regardless of previous exposure to stressors, showed higher secretion of CORT in response to LPS treatment, suggesting that previous exposure to CVS does not alter the HPA axis response to the bacterial endotoxin injection. The effects of chronic stressors on the HPA axis responsiveness are varied, with some studies showing sensitization [19, 20, 62], attenuation [21, 22], or no change [25] of the HPA response to a novel stressor. These inconsistencies indicate that the intrinsic features of the paradigms (e.g., stressors nature; duration of the protocol) might determine the direction of the HPA axis response to subsequent stressors. For instance, while chronic cold exposure sensitizes both CORT and adrenocorticotropic hormone (ACTH) responses to a subsequent immune or physical stressor [19, 20, 62], repeated exposure to mild stressors or restraint induce similar CORT increase in stress- and non-stressed rodents after an LPS challenge [21, 25].

In the current study, CVS-exposed mice showed attenuated CORT levels when compared to non-stressed animals after LPS injection. This result corroborates previous findings from our group using the same paradigm [32] and suggests that 6 weeks of CVS blunts the HPA axis reactivity of both female and male mice. We also observed sex differences in CORT levels, and female mice secreted more CORT than their male counterparts. This finding is in line with previous studies reporting higher baseline and stress-induced CORT levels in female than male rodents [28, 63]. However, it is important to emphasize that this effect is not always observed [32, 40], suggesting that the paradigm to which animals are exposed, as well as the nature of the challenge stressor (immunological vs. physical; novel vs. familiar) may alter the pattern of response of the HPA axis.

Though corticosteroids have a well-established anti-inflammatory effect in clinical settings, they can also enhance stress-induced inflammatory processes under some circumstances [64]. For instance, when administered one hour after LPS treatment, CORT mitigates the LPS-induced increase in IL-1β mRNA. In contrast, administering CORT either 2 or 24 hours before LPS does not attenuate the LPS-induced rise in IL-1β mRNA in the hippocampus [15]. Despite appearing counterintuitive, these findings imply that not only low levels of glucocorticoids create a favorable pro-inflammatory environment. In the current study, the CVS protocol does not stimulate CORT secretion, and mice exposed to CVS and injected with saline exhibited CORT levels comparable to those of non-stressed animals injected with saline and animals that were not exposed to any injection or stressor (baseline mice). These results suggest that CORT concentrations before LPS were low, potentially establishing a favorable environment for a pro-inflammatory response upon exposure to LPS.

This study sought to provide an analysis of the immune consequences of chronic stressors by assessing three pro-inflammatory markers in the plasma and brain in both male and female mice. Despite that effort, our study also has limitations. First, our endocrine and cytokine assessments are restricted to a single time-point (i.e., 2 hours after LPS injection), allowing a temporally limited analysis of the effects of stress and the bacterial endotoxin treatment. Previous studies in rodents suggest that stress exposure sensitizes, attenuates, or has no impact on plasma and brain cytokines levels in a time-dependent manner [11, 20]. Thus, to uncover whether the immune consequences of CVS in both sexes are sustained or may have a delayed emergence, future studies should include more time-points after the LPS challenge. Our findings on the brain pro-inflammatory cytokine profile point out to a region, stress, and sex dependent patterns. Microglia are the major immune cells in the brain and chronic stressors are known for inducing morphological and functional alterations in these cells [21, 23]. Additionally, previous evidence suggests that glucocorticoids exert their neuroinflammatory priming effects on male rats by acting on the microglia [13]. In future studies, it might be interesting to assess whether the cytokine findings described here are linked to microglia changes in the assessed brain regions.

In conclusion, the present results reveal limited sex differences on the pro-inflammatory consequences of chronic stress in the periphery and in the brain. Despite the lack of effect of chronic stress exposure on the pro-inflammatory response of both male and female mice, the current CVS protocol blunts the HPA reactivity to the acute immune challenge, indicating it exerts a modulatory effect on the stress axis responsiveness.

## Supporting information

S1 Fig Baseline corticosterone levels. (TIF)

S1 Table Data support document updated 1.22.2024. (XLSX)

## References

[1] McEwen BS, Akil H. Revisiting the Stress Concept: Implications for Affective Disorders. Journal of Neuroscience. 2020 Jan 2;40(1):12–21. doi: 10.1523/JNEUROSCI.0733-19.2019 31896560 PMC6939488

[2] Armario A, Restrepo C, Castellanos JM, Balasch J. Dissociation between adrenocorticotropin and corticosterone responses to restraint after previous chronic exposure to stress. Life sciences. 1985 Jun 3;36(22):2085–92. doi: 10.1016/0024-3205(85)90304-2 2987634

[3] Herman JP, Adams D, Prewitt C. Regulatory changes in neuroendocrine stress-integrative circuitry produced by a variable stress paradigm. Neuroendocrinology. 1995 Apr 9;61(2):180–90. doi: 10.1159/000126839 7753337

[4] Steptoe A, Kivimäki M. Stress and cardiovascular disease. Nature Reviews Cardiology. 2012 Jun;9(6):360–70. doi: 10.1038/nrcardio.2012.45 22473079

[5] Chandola T, Brunner E, Marmot M. Chronic stress at work and the metabolic syndrome: prospective study. Bmj. 2006 Mar 2;332(7540):521–5. doi: 10.1136/bmj.38693.435301.80 16428252 PMC1388129

[6] Kendler KS, Karkowski LM, Prescott CA. Causal relationship between stressful life events and the onset of major depression. American journal of psychiatry. 1999 Jun 1;156(6): 837–41. doi: 10.1176/ajp.156.6.837 10360120

[7] Zorn JV, Schür RR, Boks MP, Kahn RS, Joëls M, Vinkers CH. Cortisol stress reactivity across psychiatric disorders: A systematic review and meta-analysis. Psychoneuroendocrinology. 2017 Mar 1;77:25–36. doi: 10.1016/j.psyneuen.2016.11.036 28012291

[8] Cain DW, Cidlowski JA. Immune regulation by glucocorticoids. Nature Reviews Immunology. 2017 Apr;17(4):233–47. doi: 10.1038/nri.2017.1 28192415 PMC9761406

[9] O’Connor KA, Johnson JD, Hansen MK, Wieseler Frank JL, Maksimova E, Watkins LR, et al. Peripheral and central proinflammatory cytokine response to a severe acute stressor. Brain research. 2003 Nov 21;991(1–2):123–32. doi: 10.1016/j.brainres.2003.08.006 14575884

[10] Hueston CM, Deak T. The inflamed axis: the interaction between stress, hormones, and the expression of inflammatory-related genes within key structures comprising the hypothalamic-pituitary-adrenal axis. Physiology & behavior. 2014 Jan 30;124: 77–91. doi: 10.1016/j.physbeh.2013.10.035 24184413

[11] JD Johnson, KA O’Connor, T Deak, M Stark, LR Watkins, SF Maier. Prior stressor exposure sensitizes LPS-induced cytokine production. Brain, behavior, and immunity. 2002 Aug 1;16(4): 461–76. doi: 10.1006/brbi.2001.0638 12096891

[12] FrankMG, BarattaMV, SprungerDB, WatkinsLR, MaierSF. Microglia serve as a neuroimmune substrate for stress-induced potentiation of CNS pro-inflammatory cytokine responses. Brain Behav Immun. 2007 Jan;21(1):47–59. doi: 10.1016/j.bbi.2006.03.005 16647243

[13] Frank MG, Thompson BM, Watkins LR, Maier SF. Glucocorticoids mediate stress-induced priming of microglial pro-inflammatory responses. Brain Behav Immun. 2012 Feb;26(2):337–45. doi: 10.1016/j.bbi.2011.10.005 22041296 PMC5652300

[14] Fonken LK, Weber MD, Daut RA, Kitt MM, Frank MG, Watkins LR, et al. Stress-induced neuroinflammatory priming is time of day dependent. Psychoneuroendocrinology. 2016 Apr;66:82–90. doi: 10.1016/j.psyneuen.2016.01.006 26799851 PMC4788538

[15] Frank MG, Miguel ZD, Watkins LR, Maier SF. Prior exposure to glucocorticoids sensitizes the neuroinflammatory and peripheral inflammatory responses to E. coli lipopolysaccharide. Brain Behav Immun. 2010 Jan;24(1):19–30. doi: 10.1016/j.bbi.2009.07.008 19647070

[16] Fonken LK, Frank MG, Gaudet AD, D’Angelo HM, Daut RA, Hampson EC, et al. Neuroinflammatory priming to stress is differentially regulated in male and female rats. Brain Behav Immun. 2018 May;70:257–267. doi: 10.1016/j.bbi.2018.03.005 29524458 PMC5953809

[17] Mormède C, Castanon N, Médina C, Moze E, Lestage J, Neveu PJ, et al. Chronic mild stress in mice decreases peripheral cytokine and increases central cytokine expression independently of IL-10 regulation of the cytokine network. Neuroimmunomodulation. 2002 Aug 15;10(6):359–66. doi: 10.1159/000071477 12907843

[18] Zhao X, Cao F, Liu Q, Li X, Xu G, Liu G, et al. Behavioral, inflammatory and neurochemical disturbances in LPS and UCMS-induced mouse models of depression. Behavioral brain research. 2019 May 17;364:494–502. doi: 10.1016/j.bbr.2017.05.064 28572058

[19] Ma S, Morilak DA. Chronic intermittent cold stress sensitises the hypothalamic-pituitary-adrenal response to a novel acute stress by enhancing noradrenergic influence in the rat paraventricular nucleus. Journal of neuroendocrinology. 2005 Nov;17(11):761–9. doi: 10.1111/j.1365-2826.2005.01372.x 16219005

[20] Girotti M, Donegan JJ, Morilak DA. Chronic intermittent cold stress sensitizes neuro-immune reactivity in the rat brain. Psychoneuroendocrinology 2011 Sep 1:36(8): 1164–74. doi: 10.1016/j.psyneuen.2011.02.008 21411230 PMC3130087

[21] Smith BL, Schmeltzer SN, Packard BA, Sah R, Herman JP. Divergent effects of repeated restraint versus chronic variable stress on prefrontal cortical immune status after LPS injection. Brain, behavioral, and immunity. 2016 Oct 1;57:263–70. doi: 10.1016/j.bbi.2016.05.004 27177449 PMC5015433

[22] Audet MC, Jacobson-Pick S, Wann BP, Anisman H. Social defeat promotes specific cytokine variations within the prefrontal cortex upon subsequent aggressive or endotoxin challenges. Brain, behavior, and immunity. 2011 Aug 1;25(6):1197–205. doi: 10.1016/j.bbi.2011.03.010 21435391

[23] Farooq RK, Isingrini E, Tanti A, Le Guisquet AM, Arlicot N, Minier F, et al. Is unpredictable chronic mild stress (UCMS) a reliable model to study depression-induced neuroinflammation?. Behavioral brain research. 2012 May 16:231(1): 130–7. doi: 10.1016/j.bbr.2012.03.020 22465167

[24] Voorhees JL, Tarr AJ, Wohleb ES, Godbout JP, Mo X, Sheridan JF, et al. Prolonged restraint stress increases IL-6, reduces IL-10, and causes persistent depressive-like behavior that is reversed by recombinant IL-10. PloS one. 2013 Mar 8;8(3):e58488. doi: 10.1371/journal.pone.0058488 23520517 PMC3592793

[25] Couch Y, Trofimov A, Markova N, Nikolenko V, Steinbusch HW, Chekhonin V, et al. Low-dose lipopolysaccharide (LPS) inhibits aggressive and augments depressive behaviours in a chronic mild stress model in mice. Journal of neuroinflammation. 2016 Dec;13:1–7. doi: 10.1186/s12974-016-0572-0 27184538 PMC4867526

[26] Medina-Rodriguez EM, Rice KC, Jope RS, Beurel E. Comparison of inflammatory and behavioral responses to chronic stress in female and male mice. Brain, behavior, and immunity. 2022 Nov 1;106:180–97. doi: 10.1016/j.bbi.2022.08.017 36058417 PMC9561002

[27] Critchlow V, Liebelt RA, Bar-Sela M, Mountcastle W, Lipscomb HS. Sex difference in resting pituitary-adrenal function in the rat. American Journal of Physiology-Legacy Content. 1963 Nov 1;205(5):807–15. doi: 10.1152/ajplegacy.1963.205.5.807 4291060

[28] Tinnikov AA. Responses of serum corticosterone and corticosteroid-binding globulin to acute and prolonged stress in the rat. Endocrine. 1999 Oct;11(2):145–50. doi: 10.1385/ENDO:11:2:145 10709761

[29] Harpaz I, Abutbul S, Nemirovsky A, Gal R, Cohen H, Monsonego A. Chronic exposure to stress predisposes to higher autoimmune susceptibility in C57BL/6 mice: glucocorticoids as a double-edged sword. European journal of immunology. 2013 Mar;43(3):758–69. doi: 10.1002/eji.201242613 23255172

[30] Handa RJ, Burgess LH, Kerr JE, O’Keefe JA. Gonadal steroid hormone receptors and sex differences in the hypothalamo-pituitary-adrenal axis. Hormones and behavior. 1994 Dec 1:28(4): 464–76. doi: 10.1006/hbeh.1994.1044 7729815

[31] Borrow AP, Bales NJ, Stover SA, Handa RJ. Chronic Variable Stress Induces Sex-Specific Alterations in Social Behavior and Neuropeptide Expression in the Mouse. Endocrinology. 2018 Jul;159(7):2803–14. doi: 10.1210/en.2018-00217 29788320 PMC6692887

[32] Miller L, Careaga MB, Handa RJ, Wu TJ. The Effects of Chronic Variable Stress and Photoperiod Alteration on the Hypothalamic-Pituitary-Adrenal Axis Response and Behavior of Mice. Neuroscience. 2022 Aug 1;496:105–18. doi: 10.1016/j.neuroscience.2022.06.011 35700818

[33] McLean CP, Asnaani A, Litz BT, Hofmann SG. Gender differences in anxiety disorders: prevalence, course of illness, comorbidity and burden of illness. Journal of psychiatric research. 2011 Aug 1;45(8):1027–35. doi: 10.1016/j.jpsychires.2011.03.006 21439576 PMC3135672

[34] Babb JA, Masini CV, Day HE, Campeau S. Habituation of hypothalamic-pituitary-adrenocortical axis hormones to repeated homotypic stress and subsequent heterotypic stressor exposure in male and female rats. Stress. 2014 May;17(3):224–34. doi: 10.3109/10253890.2014.905534 24635729 PMC8162918

[35] Willner P, Towell A, Sampson D, Sophokleous S, Muscat R. Reduction of sucrose preference by chronic unpredictable mild stress, and its restoration by a tricyclic antidepressant. Psychopharmacology (Berl). 1987;93(3):358–64. doi: 10.1007/BF00187257 3124165

[36] Strekalova T, Spanagel R, Bartsch D, Henn FA, Gass P. Stress-induced anhedonia in mice is associated with deficits in forced swimming and exploration. Neuropsychopharmacology. 2004 Nov;29(11):2007–17. doi: 10.1038/sj.npp.1300532 15266352

[37] Cotella EM, Gómez AS, Lemen P, Chen C, Fernández G, Hansen C, et al. Long-term impact of chronic variable stress in adolescence versus adulthood. Prog Neuropsychopharmacol Biol Psychiatry. 2019 Jan 10;88:303–310. doi: 10.1016/j.pnpbp.2018.08.003 30096330 PMC6165677

[38] Aguilera G. Regulation of pituitary ACTH secretion during chronic stress. Front Neuroendocrinol. 1994 Dec;15(4):321–50. doi: 10.1006/frne.1994.1013 7895891

[39] Borrow AP, Heck AL, Miller AM, Sheng JA, Stover SA, Daniels RM, et al. Chronic variable stress alters hypothalamic-pituitary-adrenal axis function in the female mouse. Physiology & behavior. 2019 Oct 1;209:112613. doi: 10.1016/j.physbeh.2019.112613 31299374 PMC6693655

[40] Nair BB, Khant Aung Z, Porteous R, Prescott M, Glendining KA, Jenkins DE, et al. Impact of chronic variable stress on neuroendocrine hypothalamus and pituitary in male and female C57BL/6J mice. Journal of neuroendocrinology. 2021 May;33(5):e12972. doi: 10.1111/jne.12972 33896057

[41] Flak JN, Ostrander MM, Tasker JG, Herman JP. Chronic stress-induced neurotransmitter plasticity in the PVN. J Comp Neurol. 2009 Nov 10;517(2):156–65. doi: 10.1002/cne.22142 19731312 PMC4539130

[42] Carvalho-Netto EF, Myers B, Jones K, Solomon MB, Herman JP. Sex differences in synaptic plasticity in stress-responsive brain regions following chronic variable stress. Physiology & behavior. 2011 Aug 3;104(2):242–247. doi: 10.1016/j.physbeh.2011.01.024 21315096 PMC4486019

[43] Turner MD, Nedjai B, Hurst T, Pennington DJ. Cytokines and chemokines: At the crossroads of cell signalling and inflammatory disease. Biochimica et Biophysica Acta (BBA)-Molecular Cell Research. 2014 Nov 1;1843(11):2563–82. doi: 10.1016/j.bbamcr.2014.05.014 24892271

[44] Munhoz CD, Lepsch LB, Kawamoto EM, Malta MB, Lima Lde S, Avellar MC, et al. Chronic unpredictable stress exacerbates lipopolysaccharide-induced activation of nuclear factor-kappaB in the frontal cortex and hippocampus via glucocorticoid secretion. J Neurosci. 2006 Apr 5;26(14):3813–20. doi: 10.1523/JNEUROSCI.4398-05.2006 16597735 PMC6674142

[45] Everhardt Queen A, Moerdyk-Schauwecker M, McKee LM, Leamy LJ, Huet YM. Differential expression of inflammatory cytokines and stress genes in male and female mice in response to a lipopolysaccharide challenge. PloS one. 2016 Apr 27;11(4):e0152289. doi: 10.1371/journal.pone.0152289 27120355 PMC4847773

[46] Keith BJ, Franklin GP, Paxinos G. The mouse brain in stereotaxic coordinates. California: Academic. 2008.

[47] Ulrich-Lai YM, Figueiredo HF, Ostrander MM, Choi DC, Engeland WC, Herman JP. Chronic stress induces adrenal hyperplasia and hypertrophy in a subregion-specific manner. Am J Physiol Endocrinol Metab. 2006 Nov;291(5):E965–73. doi: 10.1152/ajpendo.00070.2006 16772325

[48] Heck AL, Sheng JA, Miller AM, Stover SA, Bales NJ, Tan SML, et al. Social isolation alters hypothalamic pituitary adrenal axis activity after chronic variable stress in male C57BL/6 mice. Stress. 2020 Jul 3;23(4): 457–65. doi: 10.1080/10253890.2020.1733962 32093522 PMC7376957

[49] Solomon MB, Jankord R, Flak JN, Herman JP. Chronic stress, energy balance and adiposity in female rats. Physiology & behavior. 2011 Jan 10;102(1):84–90. doi: 10.1016/j.physbeh.2010.09.024 20932852 PMC3991931

[50] Wagner KV, Wang XD, Liebl C, Scharf SH, Müller MB, Schmidt MV. Pituitary glucocorticoid receptor deletion reduces vulnerability to chronic stress. Psychoneuroendocrinology. 2011 May 1;36(4):579–87. doi: 10.1016/j.psyneuen.2010.09.007 20940090

[51] van Doeselaar L, Yang H, Bordes J, Brix L, Engelhardt C, Tang F, et al. Chronic social defeat stress in female mice leads to sex-specific behavioral and neuroendocrine effects. Stress. 2021 Mar 4;24(2):168–80. doi: 10.1080/10253890.2020.1864319 33322989

[52] Pankevich DE, Bale TL. Stress and sex influences on food-seeking behaviors. Obesity. 2008 Jul;16(7):1539–44. doi: 10.1038/oby.2008.221 18421275

[53] Simpkiss JL, Devine DP. Responses of the HPA axis after chronic variable stress: effects of novel and familiar stressors. Neuroendocrinology Letters. 2003 Feb 1;24(1–2):97–103. 12743542

[54] Menees KB, Earls RH, Chung J, Jernigan J, Filipov NM, Carpenter JM, et al. Sex- and age-dependent alterations of splenic immune cell profile and NK cell phenotypes and function in C57BL/6J mice. Immun Ageing. 2021 Jan 8;18(1):3. doi: 10.1186/s12979-021-00214-3 33419446 PMC7791703

[55] Laukova M, Vargovic P, Rokytova I, Manz G, Kvetnansky R. Repeated Stress Exaggerates Lipopolysaccharide-Induced Inflammatory Response in the Rat Spleen. Cellular and Molecular Neurobiology. 2018 Jan;38(1):195–208. doi: 10.1007/s10571-017-0546-5 28884416 PMC11481850

[56] de Kloet ER, Joëls M, Holsboer F. Stress and the brain: from adaptation to disease. Nature reviews neuroscience. 2005 Jun 1;6(6): 463–75. doi: 10.1038/nrn1683 15891777

[57] Herman JP, Tasker JG. Paraventricular Hypothalamic Mechanisms of Chronic Stress Adaptation. Frontiers in endocrinology. 2016. Oct 31;7:137. doi: 10.3389/fendo.2016.00137 27843437 PMC5086584

[58] Vallières L, Rivest S. Regulation of the genes encoding interleukin-6, its receptor, and gp130 in the rat brain in response to the immune activator lipopolysaccharide and the proinflammatory cytokine interleukin-1beta. Journal of neurochemistry. 1997 Oct;69(4):1668–83. doi: 10.1046/j.1471-4159.1997.69041668.x 9326296

[59] Gruol DL. IL-6 regulation of synaptic function in the CNS. Neuropharmacology. 2015 Sep;96(Pt A):42–54. doi: 10.1016/j.neuropharm.2014.10.023 25445486 PMC4446251

[60] Bialuk I, Taranta A, Winnicka MM. IL-6 deficiency alters spatial memory in 4- and 24-month-old mice. Neurobiol Learn Mem. 2018 Nov;155:21–29. doi: 10.1016/j.nlm.2018.06.006 29908286

[61] Kemp GM, Altimimi HF, Nho Y, Heir R, Klyczek A, Stellwagen D. Sustained TNF signaling is required for the synaptic and anxiety-like behavioral response to acute stress. Mol Psychiatry. 2022 Nov;27(11):4474–4484. doi: 10.1038/s41380-022-01737-x 36104437 PMC9734040

[62] Pardon MC, Ma S, Morilak DA. Chronic cold stress sensitizes brain noradrenergic reactivity and noradrenergic facilitation of the HPA stress response in Wistar Kyoto rats. Brain research. 2003 May 2;971(1):55–65. doi: 10.1016/s0006-8993(03)02355-2 12691837

[63] Solomon MB, Loftspring M, de Kloet AD, Ghosal S, Jankord R, Flak JN, et al. Neuroendocrine Function After Hypothalamic Depletion of Glucocorticoid Receptors in Male and Female Mice. Endocrinology. 2015 Aug 1;156(8):2843–53. doi: 10.1210/en.2015-1276 26046806 PMC4511133

[64] Sorrells SF, Sapolsky RM. An inflammatory review of glucocorticoid actions in the CNS. Brain, behavior, and immunity. 2007 Mar 1;21(3):259–72. doi: 10.1016/j.bbi.2006.11.006 17194565 PMC1997278

