# Supplementary figures and images for: Chronically stressed male and female mice show a similar peripheral and central pro-inflammatory profile after an immune challenge
Source: PLoS One. 2024 Feb 21;19(2):e0297776. doi: 10.1371/journal.pone.0297776 (PMC10880960; doi:10.1371/journal.pone.0297776)

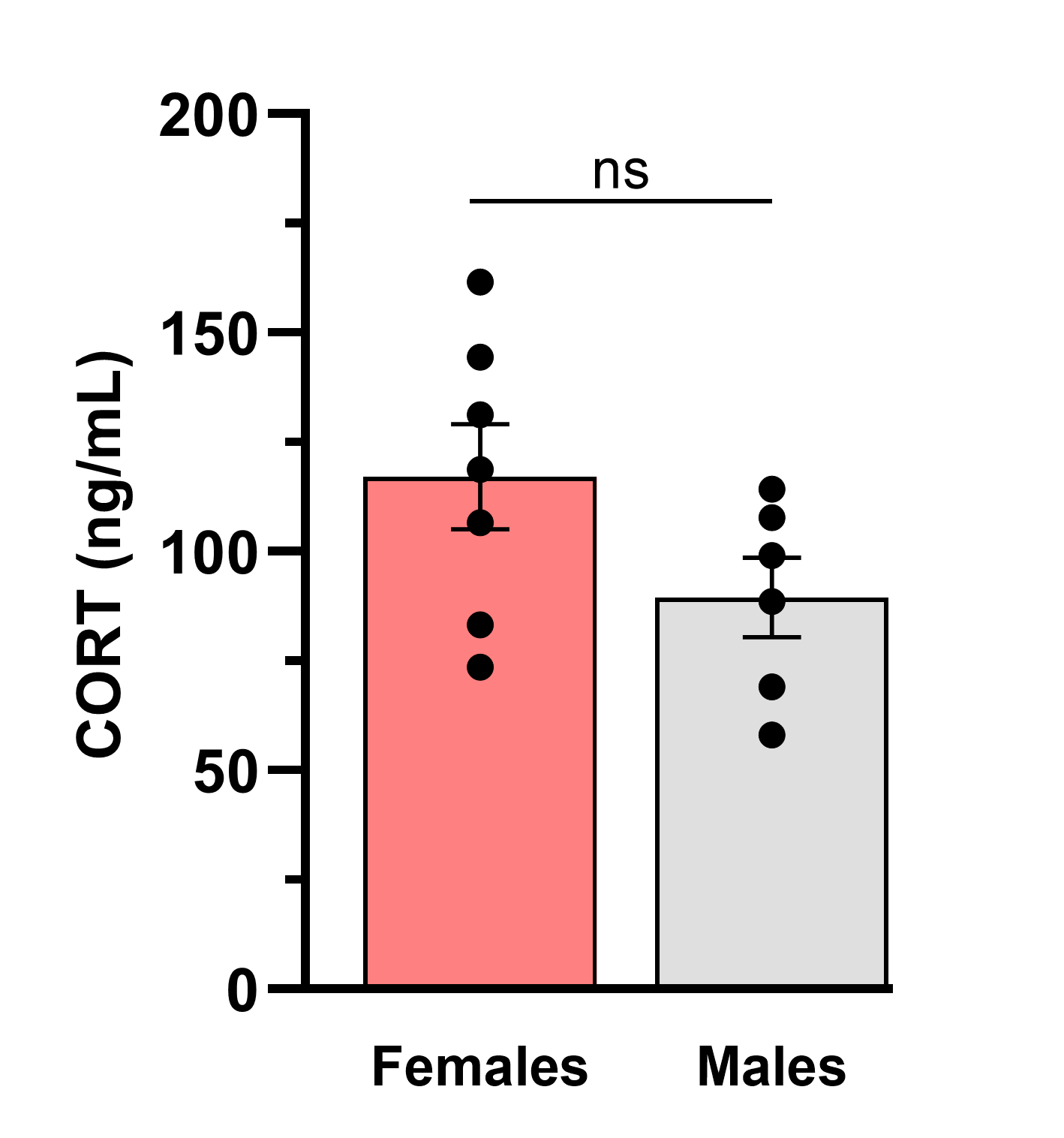

Supplement: S1 Fig — (TIF) [file pone.0297776.s001.tif]
